# Investigation of the gut parasitic community composition in *Manis pentadactyla* and *Manis javanica* based on high-throughput amplicon sequencing

**DOI:** 10.3389/fvets.2025.1712988

**Published:** 2025-12-15

**Authors:** Sitong Chen, Xianghe Wang, Xinyu Liu, Meiling Xie, Fuyu An, Kai Wang, Zhenquan Zhang, Shuo Zhou, Yan Hua

**Affiliations:** 1 College of Life Sciences, Zhongkai University of Agriculture and Engineering, Guangzhou, China; 2 Guangdong Provincial Key Laboratory of Silviculture, Protection and Utilization, Guangdong Academy of Forestry, Guangzhou, China; 3 Guangdong Provincial Key Laboratory of Zoonosis Prevention and Control, College of Veterinary Medicine, South China Agricultural University, Guangzhou, China

**Keywords:** *Manis pentadactyla*, *Manis javanica*, 18S rRNA, potentially pathogenic parasites, wildlife

## Abstract

**Introduction:**

The critically endangered *Manis pentadactyla* and *Manis javanica* are increasingly threatened by parasitic infections, with gastrointestinal nematodes and protozoan parasites particularly prominent.

**Methods:**

In this study, 72 fecal samples were collected from captive *M. javanica* and *M. pentadactyla* housed at the Guangdong Wildlife Monitoring and Rescue Center in China. High-throughput amplicon sequencing targeting the V9 region of the 18S rRNA gene was employed to investigate the community composition and diversity of their gastrointestinal parasite communities.

**Results:**

Our results revealed significant differences in the composition of parasitic communities across species and sex groups. Several potentially pathogenic helminths and protozoa were identified. The helminths included Rhabditida, Moniliformida, and Diplogasterida. Protozoan taxa such as *Eimeria*, *Cryptosporidium*, and other free-living protozoa such as *Acanthamoeba*, *Colpoda*, *Vermamoeba*, *Bicosoecida*, and *Trinema* were found to be highly abundant and widely distributed. While these free-living protozoa are commonly found in environmental samples, there is no solid evidence of their parasitic or pathogenic role in pangolins.

**Discussion:**

This study systematically characterized the gastrointestinal parasitic communities in captive *M. pentadactyla* and *M. javanica*. Notably, Apicomplexa and Nematoda were found to be dominant and likely represented the most susceptible parasitic groups in pangolins under captive conditions. These findings offer valuable insights for the diagnosis, treatment, and health management of parasitic infections in pangolin conservation.

## 1 Introduction

The pangolin, classified under Mammalia, order Pholidota, family Manidae, and genus *Manis*, is one of the most heavily trafficked mammals in the global illegal wildlife trade (1, 2). All eight species of pangolin currently recognized have been classified as Critically Endangered by the IUCN (3). Among them, *Manis pentadactyla* and *Manis javanica* are the primary species distributed in China (4). Since 2020, the species has been included in the *List of Fauna under Special State Protection* (LFSSP) and designated as a national first-class protected animal in China, as part of enhanced efforts to strengthen its conservation (5). Since then, efforts to rescue wild individuals and manage captive breeding have been progressively intensified, encompassing clinical treatment, husbandry management, and population recovery of pangolins (6). However, rescue and captive environments have also disrupted the species’ natural ecological barriers (7); these changes have increased pangolins’ exposure to pathogens, heightening their susceptibility to health risks, most notably, the growing concern over infectious diseases (8). Current research indicates that pangolins are susceptible to various pathogens, including viruses, bacteria, and parasites (9). Among these, parasitic infections-particularly those caused by gastrointestinal parasites-have attracted considerable attention.

Gastrointestinal nematodes are among the most prevalent parasite groups in wild mammals, with over 10,500 species documented in rodents alone (10). Their primary transmission route is fecal-oral contact, which represents one of the most common modes of infection among wild animals (11). Due to their unique ecological habits and feeding behaviors, pangolins are highly susceptible to gastrointestinal parasitic infections when they come into contact with contaminated soil, water, or food sources. Such infections can lead to clinical symptoms including anemia, emaciation, and immunosuppression, and may result in mortality in severe cases (12, 13). According to Wicker et al. (14), recent field rescues of pangolins have revealed a significantly high prevalence of parasitic infections in these animals. This underscores the importance of rigorous parasitic surveillance combined with timely and targeted treatment, which can reduce both the transmission risk and mortality associated with parasitic infections in captive pangolin populations (15).

In current research practices, non-invasive fecal sampling has become a practical approach for monitoring parasitic infections in wild mammals. This method provides valuable insights into host and gastrointestinal parasite dynamics and minimizes animal disturbance (16). Conventional microscopic examination typically identifies specific parasite ova; however, it is limited by low sensitivity and an inability to detect latent or mixed infections (17). In contrast, High-throughput amplicon sequencing technology offers a powerful and efficient way of assessing parasitic disease diversity (18). This approach enables the detection of parasites in their latent stages and accurately identifies mixed infections.

In this study, we employed high-throughput amplicon sequencing to systematically analyze the composition and diversity of gastrointestinal parasites in both *M. pentadactyla* and *M. javanica*. Notably, while Nematoda and Apicomplexa were the primary focus of our investigation, we also detected non-parasitic, free-living eukaryotes such as *Acanthamoeba* and *Colpoda*. These organisms, although present in the samples, do not qualify as parasitic, as they typically exist independently in the environment and only potentially interact with the host under certain conditions (19). This distinction is crucial for accurately interpreting the results and understanding the ecological relationships within the gastrointestinal microbiota of pangolins. This study aims to provide foundational data for health assessments and evidence-based deworming strategies, supporting future conservation and rescue efforts for these endangered species.

## 2 Materials and methods

### 2.1 Sample collection

In September 2023, the research team collected a total of 72 fecal samples from captive pangolins at the Guangdong Wildlife Monitoring and Rescue Center, including 26 samples from *M. pentadactyla* and 46 samples from *M. javanica*. The sample collection process was meticulously designed to ensure both representativeness and independence. Each pangolin was housed individually in dedicated enclosures, with strict protocols for disinfecting the entire body and changing gloves and shoe covers before entering or exiting each enclosure. All pangolins were handled according to standardized husbandry practices to minimize stress and reduce the risk of pathogen transmission, while ensuring the integrity of the samples. All pangolins were individually housed in specialized enclosures, with only one individual per enclosure, to minimize stress and the potential transmission of pathogens, while ensuring sample integrity. The fecal samples collected were from rescued pangolins, and none of these individuals had received deworming treatment before sampling. Fecal samples were collected within 30 min of spontaneous defecation during the night, as pangolins are nocturnal, to ensure sample freshness and minimize cross-contamination risks. The entire collection process strictly adhered to hygiene protocols, using disposable gloves, sterile centrifuge tubes, and sterile cotton swabs to prevent contamination. The collected samples were immediately placed in foam boxes with ice packs and transported to the laboratory within 2 h. In the laboratory, all samples were stored at −80 °C until further analysis via high-throughput amplicon sequencing. To minimize stress on the animals, the sampling process was conducted quietly and without direct contact with the animals.

### 2.2 DNA extraction and PCR amplification

Genomic DNA was extracted from all samples using the MagaBio Soil/Feces Genomic DNA Purification Kit, following the manufacturer’s instructions. The purity and concentration of the extracted DNA were assessed using a NanoDrop One spectrophotometer (Thermo Fisher Scientific, MA, United States). The extracted DNA was stored at −80 °C for long-term preservation and subsequent PCR amplification. PCR amplification was performed using a Bio-Rad S1000 thermal cycler (Bio-Rad Laboratories, CA, United States). Using genomic DNA as the template, the V9 region of the 18S rRNA gene, suitable for profiling eukaryotic microbial diversity, was amplified with barcode-tagged specific primers and Premix Taq polymerase. The specific primers used for amplification were NF1 (5′-GGTGGTGCATGGCCGTTCTTAGTT-3′) and 18Sr2b (5′-TACAAAGGGCAGGGACGTAAT-3′). All primers were synthesized by Sangon Biotech (Shanghai, China). It is important to note that the 18S rRNA gene is specifically applicable to confirmed parasitic taxa, such as Apicomplexa and Nematoda, and is not suitable for all eukaryotic organisms.

### 2.3 Electrophoretic analysis and gel purification of PCR products

Following concentration assessment of the PCR products using Gene Tools Analysis Software (Version 4.03.05.0, SynGene), the required volume of each sample was calculated based on equal mass principles. The PCR products were then pooled accordingly. The pooled PCR products were purified using the E.Z.N.A.^®^ Gel Extraction Kit (Omega, United States), and the target DNA fragments were eluted with TE buffer.

### 2.4 Library preparation and sequencing

Library construction was performed following the standard protocol of the ALFA-SEQ DNA Library Prep Kit. The integrity and insert size of the library fragments were assessed using the Qsep400 high-throughput nucleic acid and protein analysis system (Hangzhou Houze Biotechnology Co., Ltd., China). Libraries meeting the quality criteria were subsequently subjected to sequencing. Library concentrations were quantified using the Qubit 4.0 fluorometer (Thermo Fisher Scientific, Waltham, United States). PCR pooled samples with concentrations >8 ng/μL and a total amount of ≥0.3 μg were considered valid. Libraries that passed quality control were further assessed using the Qsep400 high-throughput nucleic acid and protein analysis system (Hangzhou Houze Biotechnology Co., Ltd., China). To pass quality control, libraries must show a prominent peak corresponding to the amplified target fragment with no adapter contamination, and the total amount must exceed 75 ng. Libraries meeting these criteria were pooled into a single flow cell based on their effective concentrations and the target sequencing data yield. After cluster generation on a cBOT system, paired-end 250 bp (PE250) sequencing of the amplicon libraries was performed on the Illumina platform (Guangdong Magigene Biotechnology Co., Ltd., Guangzhou, China).

### 2.5 Data analysis

Following sequencing, the raw paired-end reads were quality-trimmed using fastp (an ultra-fast all-in-one FASTQ preprocessor, version 0.14.1) with the following trimming parameters: -W4-M20. Primer sequences at both ends of the reads were removed using Cutadapt, based on the known primer information, resulting in high-quality, primer-free paired-end clean reads for downstream analysis. The raw reads were then subjected to quality control to obtain clean reads. After merging the paired-end reads, a second round of quality control was performed to yield clean tags, while also removing chimeric sequences. Based on the overlap between paired-end reads, USEARCH (fastq-mergepairs command) filters out non-conforming tags, generating the initial set of merged sequences (Raw Tags). Fastp was then employed to perform sliding window quality trimming on the Raw Tags (parameters: -W4-M20), resulting in high-quality merged sequences called Clean Tags. The Clean Tags were clustered into Operational Taxonomic Units (OTUs) at 97% similarity using the UPARSE clustering algorithm. The OTUs were then annotated taxonomically by aligning representative sequences against the Silva database (v132) using USEARCH v10.0.240 with a confidence threshold of 0.8. Contaminants such as mitochondria and chloroplasts were removed, along with singletons and OTUs that lacked kingdom-level annotations. Representative sequences of each OTU were aligned to reference databases using USEARCH v10.0.240 with the -sintax command for taxonomic annotation. The confidence threshold for species identification was set at 0.8. The top 20 OTUs with the highest relative abundance were selected for phylogenetic analysis. Representative sequences of these OTUs were aligned and used to construct a maximum-likelihood tree with FastTree. The resulting tree and each OTU’s relative abundance and taxonomic annotation confidence were visualized using the ggtree R package. In this study, the diversity analysis of all samples included both parasitic and non-parasitic species to provide a comprehensive overview of the eukaryotic community diversity within the samples. To ensure the scientific validity of the results, subsequent analyses will clearly delineate these two taxonomic groups. To minimize potential biases, we acknowledge the possible influence of environmental DNA and cross-contamination on high-throughput sequencing data. Stringent sterile techniques were applied throughout the sample collection, DNA extraction, PCR amplification, and library construction processes. Additionally, blank controls were incorporated at each step to rule out the presence of exogenous DNA contamination.

### 2.6 Statistical analysis

In this study, all statistical analyses were conducted using appropriate methods to ensure the accuracy and reliability of the results. Alpha diversity comparisons were performed using the Kruskal-Wallis rank-sum test or Student’s *t*-test, depending on the number of groups being compared. Pairwise comparisons for multiple groups were conducted using Dunn’s post-hoc test when necessary. For beta diversity, Principal Coordinates Analysis (PCoA) based on Bray–Curtis dissimilarity was used to visualize community structure. The statistical significance of differences in community composition between groups was assessed using Adonis analysis (permutational multivariate analysis of variance), with *R*^2^ values reported to quantify the proportion of variance explained. LEfSe (Linear Discriminant Analysis Effect Size) was employed to identify significantly abundant species across groups, with initial detection via the Kruskal-Wallis test, followed by pairwise comparisons using the Wilcoxon rank-sum test. Linear Discriminant Analysis (LDA) was then used to assess the effect size of the significantly different species, with the LDA score threshold set to ≥2 to identify biomarkers. For relative abundance comparisons, the Kruskal-Wallis test (for three or more groups) or Student’s *t*-test (for two groups) was applied. For multiple comparisons, False Discovery Rate (FDR) correction was used to reduce the occurrence of false positives. All bioinformatics analyses followed strict quality control standards to ensure reproducibility. The Silva database was used for taxonomic annotation, and BLAST was employed to verify the taxonomic identity of OTU representative sequences. The resulting data were analyzed using R (v4.0.5), with visualization and further statistical analysis conducted using the ggplot2, ggtree, and vegan R packages.

## 3 Results

We sequenced the 18S V9 region from 72 samples, generating approximately 8.382 GB of raw reads. The average number of reads per sample was 0.118 GB, with the maximum and minimum read volumes per sample being 0.1366 GB and 0.0605 GB, respectively.

### 3.1 Community composition of parasites

This study analyzed parasite OTUs from 72 samples across five taxonomic levels: phylum, class, order, family, and genus. Twelve phyla, 22 classes, 24 orders, 18 families, and 19 genera were identified from the fecal samples of *M. pentadactyla* and *M. javanica*.

**Figure 1:**
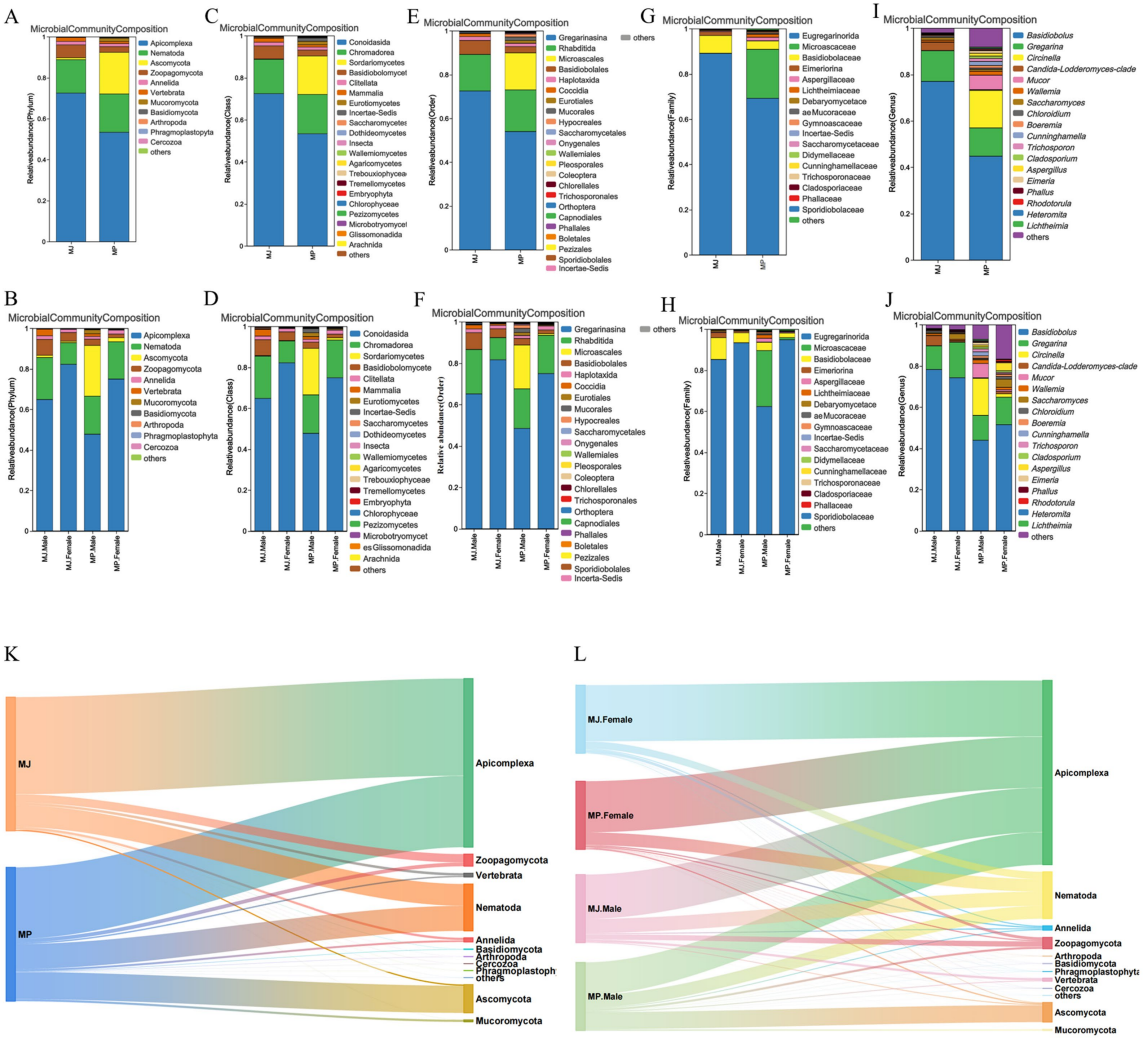
Stacked bar charts and Sankey diagrams illustrating species abundance at different taxonomic levels. **(A)** A stacked bar chart at the phylum level. **(B)** A stacked bar chart at the phylum level stratified by sex. **(C)** A stacked bar chart at the class level. **(D)** A stacked bar chart at the class level stratified by sex. **(E)** A stacked bar chart at the order level. **(F)** A stacked bar chart at the order level stratified by sex. **(G)** A stacked bar chart at the family level. **(H)** A stacked bar chart at the family level stratified by sex. **(I)** Stacked bar chart at the genus level. **(J)** A stacked bar chart at the genus level stratified by sex. **(K)** Sankey diagram depicting taxonomic relationships at the phylum level. **(L)** Sankey diagram depicting sex-specific taxonomic relationships at the phylum level.

At the phylum level, a high relative abundance of Apicomplexa and Nematoda was found in both pangolin species. In *M. javanica*, the relative abundances of Apicomplexa and Nematoda were 72.5 and 16.3%, respectively, higher than those in *M. pentadactyla* (53.4 and 18.7%) (Figure 1A). For *M. javanica*, the relative abundances of Apicomplexa and Nematoda were 64.9 and 20.7% in the male group, respectively, while they were 82.3 and 10.6% in the female group. In *M. pentadactyla*, the corresponding values were 47.7 and 18.8% for males, and 75.0 and 18.4% for females, respectively. Overall, the relative abundance of Apicomplexa was higher in *M. javanica* compared to *M. pentadactyla*, whereas the abundance of Nematoda was slightly higher in *M. pentadactyla* (Figure 1B).

At the class level, fecal samples from both *M. pentadactyla* and *M. javanica* harbored Conoidasida and Chromadorea. The relative abundance of Conoidasida in *M. javanica* was 72.4%, significantly exceeding that observed in *M. pentadactyla* (53.4%). In contrast, the relative abundance of Chromadorea exhibited minimal variation between the two species, accounting for 16.3 and 18.7%, respectively (Figure 1C). When stratified by sex, Conoidasida abundance in female *M. javanica* reached 82.2%, surpassing the 64.8% recorded in males. Conversely, Chromadorea displayed an opposite pattern, with males exhibiting a higher relative abundance (20.7%) compared to females (10.6%). A comparable trend was noted in *M. pentadactyla*, where females showed elevated Conoidasida abundance (74.9%) relative to males (47.7%), while sex-based differences in Chromadorea abundance remained minor (Figure 1D).

At the order level, both *M. pentadactyla* and *M. javanica* harbored Rhabditida and Coccidia. The relative abundance of Rhabditida in *M. javanica* was 16.6%, slightly lower than that observed in *M. pentadactyla* (19.0%). In contrast, the relative abundance of Coccidia in *M. javanica* was 1.3%, markedly higher than the 0.1% detected in *M. pentadactyla* (Figure 1E). Within *M. javanica*, males exhibited relative abundances of 21.4% for Rhabditida and 1.8% for Coccidia, whereas females showed 10.6 and 0.6%, respectively. In *M. pentadactyla*, males and females had similar relative abundances of Rhabditida (19.1 and 18.4%) and Coccidia (both 0.1%) (Figure 1F). Overall, Coccidia abundance was significantly higher in *M. javanica* than in *M. pentadactyla*, with the highest levels observed in *M. javanica* males.

At the family level, Eimeriorina was detected in both *M. pentadactyla* and *M. javanica*. The relative abundance of Eimeriorina in *M. javanica* was 1.6%, markedly higher than the 0.1% observed in *M. pentadactyla* (Figure 1G). Within *M. javanica*, males exhibited a relative abundance of 2.4%, significantly exceeding the 0.7% recorded in females. In contrast, *M. pentadactyla* males showed a relative abundance of 0.1%, which was markedly lower than the 92.8% detected in females (Figure 1H).

At the genus level, *Eimeria* was detected in both *M. pentadactyla* and *M. javanica*. The relative abundance of *Eimeria* in *M. javanica* was 79.3%, significantly higher than the 1.1% observed in *M. pentadactyla* (Figure 1I). Within *M. javanica*, males exhibited a relative abundance of 0.1%, considerably lower than the 60.5% recorded in females. Conversely, in *M. pentadactyla*, males showed a relative abundance of 1.2%, markedly exceeding the 0.1% detected in females (Figure 1J).

The constructed Sankey diagrams also illustrate the relatively broad representation of Apicomplexa and Nematoda, indicating their high abundance and status as dominant taxa within the samples (Figures 1K,L).

We observed that Apicomplexa and Nematoda, with high relative abundances, dominate the fecal samples of *M. javanica* and *M. pentadactyla*, and these taxa are confirmed as parasitic lineages. In contrast, taxa such as *Acanthamoeba*, *Colpoda*, and *Vermamoeba* are free-living eukaryotes. Despite their substantial presence in the samples, these species should not be regarded as parasites.

### 3.2 Parasite community diversity

This study employed *β*-diversity and *α*-diversity analyses to systematically investigate the compositional differences of parasite communities in fecal samples from *M. javanica* (MJ) and *M. pentadactyla* (MP). Previous studies have shown that *M. pentadactyla* weighing less than 2 kg are considered subadult, while individuals weighing more than 2 kg are classified as adults (20). All rescued *M. pentadactyla* and *M. javanica* included in this study weighed more than 2 kg, and were thus all considered adult individuals. Therefore, age was not included as a grouping variable in the analysis. The *M. javanica* group (MJ) was further subdivided into male (MJ. Male) and female (MJ. Female) subgroups, while the *M. pentadactyla* group (MP) was similarly divided into male (MP. Male) and female (MP. Female) subgroups. This stratification allowed for a more detailed examination of parasite community diversity across sexes within each pangolin species.

**Figure 2:**
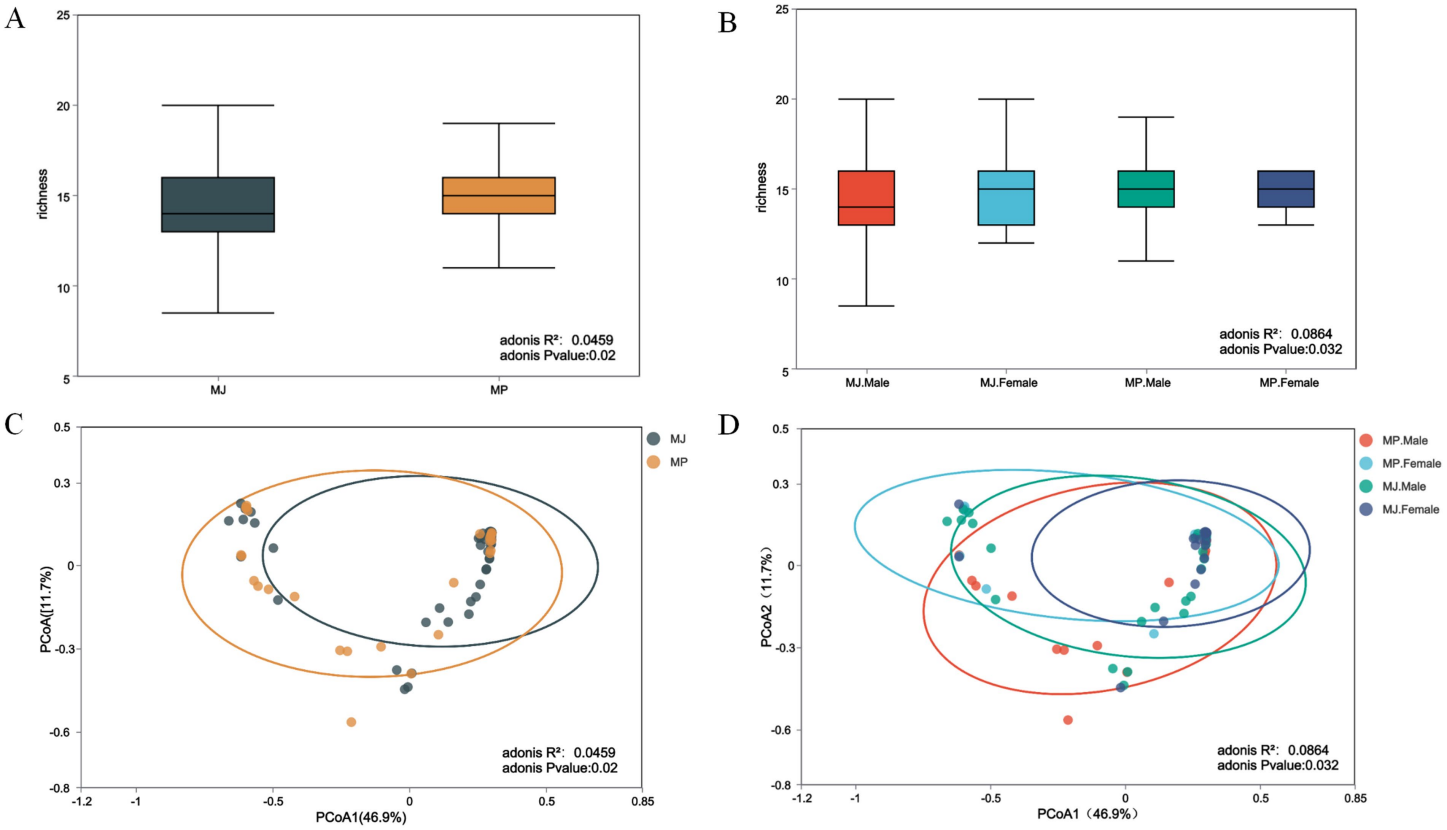
Combined *α*- and *β*-diversity analyses (*p* < 0.05). **(A)** α-diversity analysis of the two pangolin species. **(B)** α-diversity analysis stratified by sex in the two pangolin species. **(C)** β-diversity analysis of the two pangolin species. **(D)** β-diversity analysis stratified by sex in the two pangolin species.

Boxplot analysis of *α*-diversity indices revealed a significant difference in parasite community richness between the MJ and MP groups, with the median richness in the MP group exceeding that of the MJ group. Both groups exhibited similar levels of dispersion (Figure 2A). Adonis analysis indicated that the community structure differences between the MJ and MP groups were statistically significant (adonis *R*^2^ = 0.0459, *p* = 0.02). Analysis of the four sex-specific subgroups revealed distinct patterns and distributions in the richness boxplots, confirming that sex influences parasite community richness in individual pangolins (Figure 2B). Corresponding Adonis analysis further supported this difference (Adonis *R*^2^ = 0.0864, *p* = 0.032).

Principal Coordinates Analysis (PCoA) based on Bray-Curtis’s distance was performed to assess the distribution of the two populations. The first principal coordinate (PCoA1) explained 46.9% of the variance, while the second (PCoA2) accounted for 11.7%. PCoA visualization indicated a degree of clustering with partial overlap between the two groups, suggesting a high similarity of parasite communities within each group. However, a slight separation trend was also observed: samples from the MJ group were primarily clustered in the lower right region. In contrast, samples from the MP group were more dispersed, with some positioned close to the MJ cluster. This pattern indicates that the two groups share similarities yet exhibit distinct differences (Figure 2C; Adonis *R*^2^ = 0.0459, *p* = 0.02). The distribution of different sexes within the same population showed that the first principal coordinate axis (PCoA1) explained 46.9% of the variance. The second principal coordinate axis (PCoA2) accounted for 11.7% of the variance. Although partial overlap remains, samples from the four subgroups are relatively dispersed, with no clear boundaries or substantial clustering between subgroups. This distribution pattern further indicates that sex exerts a certain degree of influence on sample distribution (Figure 2D; adonis *R*^2^ = 0.0864, *p* = 0.032). The low *R*^2^ values may be due to the high variability of the parasite communities and multiple unmeasured influencing factors, such as host immune differences, environmental variables, and microbiome composition. Additionally, the heterogeneity of sample size, data distribution, and the limitations of the statistical methods may have contributed to the reduced explanatory power of the models.

### 3.3 LEfSe analysis

**Figure 3:**
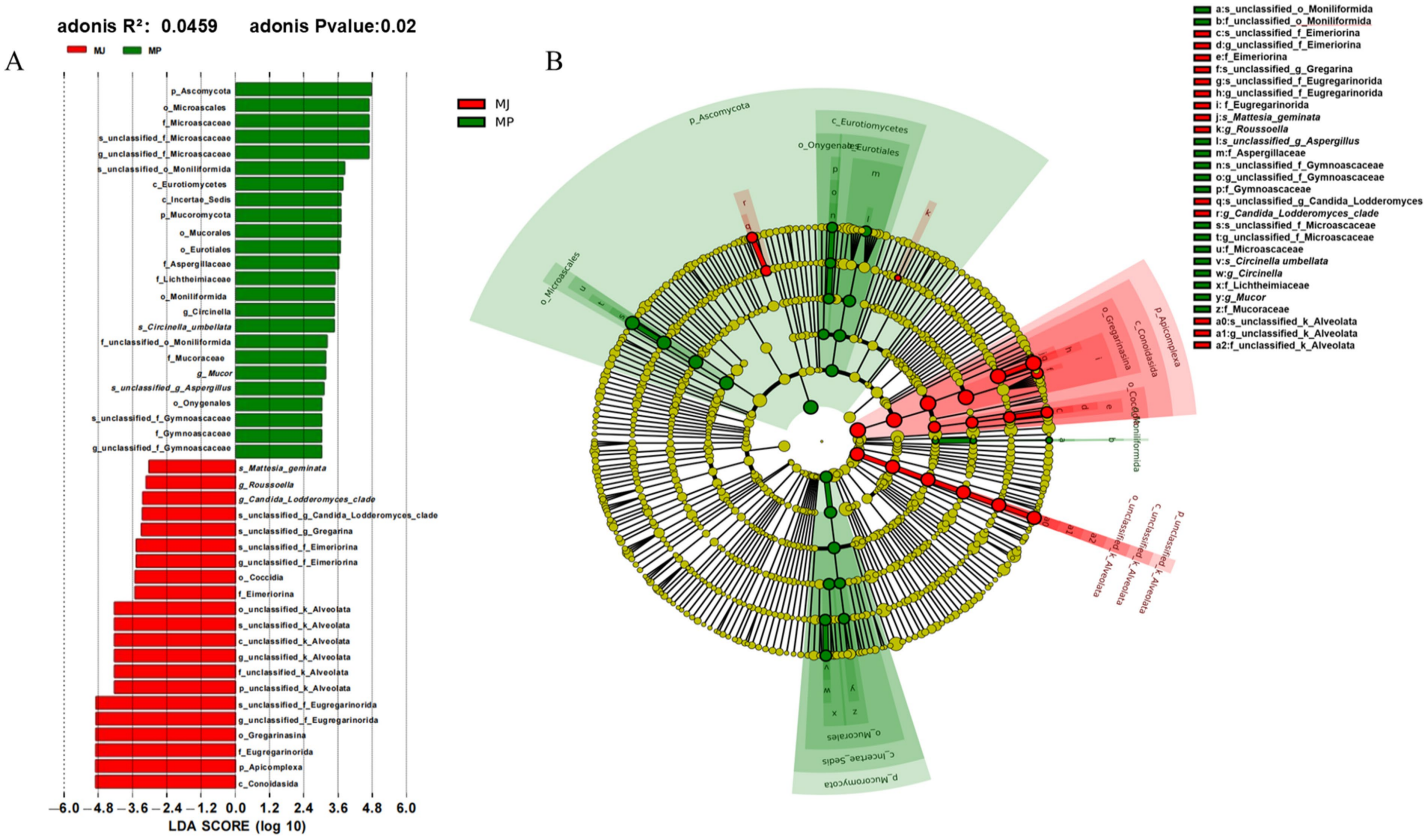
Taxa exhibiting significant differences identified by LEfSe analysis (LDA > 3, *p* < 0.05). **(A)** Linear Discriminant Analysis (LDA) bar plot. **(B)** Phylogenetic cladogram.

We applied LEfSe analysis to identify compositional differences and phylogenetic characteristics of intestinal parasites in fecal samples from *M. javanica* (MJ) and *M. pentadactyla* (MP), selecting phyla with an LDA score greater than 3 for further screening. The LDA bar plot revealed that, in addition to the significant enrichment of Aspergillaceae, Microsporidiaceae, and Mucoraceae in the fecal samples of both pangolin species, Acanthocephala, a phylum of zoonotic parasites, was detected explicitly in *M. pentadactyla*. Members of Acanthocephala attach to the small intestinal mucosa using a proboscis armed with hooks, causing mucosal congestion, hemorrhage, necrosis, and ulceration, often leading to localized endemic outbreaks. In the MJ samples, significantly enriched taxa included the order Eimeriorina, subclass Coccidia, subphylum Apicomplexa, and class Conoidasida. Both Eimeriorina and Coccidia cause coccidiosis, which can result in intestinal damage and mortality in the host (Figure 3A). Corresponding Adonis analysis demonstrated that the overall parasite community structures in fecal samples from MJ and MP differed significantly (Adonis *R*^2^ = 0.0459, *p* = 0.02), further supporting the presence of systematic differences in parasite composition between the two pangolin species. Furthermore, the phylogenetic cladogram (Figure 3B) clearly illustrates the evolutionary relationships of the differential taxa, with enriched groups in MP predominantly clustered within multiple branches of the phylum Fungi. In contrast, those enriched in MJ are mainly distributed among taxa related to the phylum Apicomplexa. These differences may be closely associated with the dietary habits, habitats, and immune response characteristics of the two pangolin species, and they also provide essential insights for assessing and controlling zoonotic parasitic diseases.

### 3.4 Analysis of potentially pathogenic parasites

**Figure 4:**
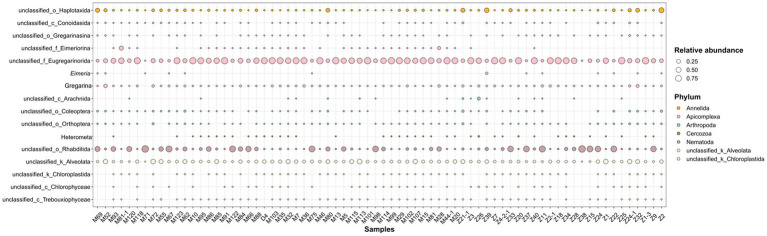
Bubble plot illustrating the relative abundance of potentially pathogenic parasites across 72 samples.

This study systematically analyzes the composition of parasites and protozoa detected in fecal samples from *M. pentadactyla* and *M. javanica*, with a particular focus on eukaryotic parasites with potential pathogenicity. The results reveal the predominant eukaryotic parasitic groups, including Nematoda (roundworms), Apicomplexa (a group of protozoa), and certain unclassified eukaryotic parasitic groups (Figure 4).

Among the identified nematode groups, the order Rhabditida was detected in the majority of samples, indicating its widespread distribution within the pangolin population. Rhabditida is a common group of parasitic nematodes found in the gastrointestinal tracts of mammals, with certain species exhibiting clear pathogenicity. These nematodes are known to cause diarrhea, intestinal inflammation, and malabsorption. As shown in the figure, the relative abundance of Rhabditida is notably high across several samples, with some samples exhibiting a relative abundance approaching 0.75. This suggests that Rhabditida may be among the most prevalent parasitic organisms in the pangolin gastrointestinal tract. These findings further support the hypothesis that pangolins may be infected with Rhabditida, potentially exhibiting corresponding clinical symptoms. The results also revealed that Apicomplexa protozoa exhibited a relatively high abundance in multiple samples, primarily including the genera *Eimeria* and Gregarina. As shown in the figure, *Eimeria* was consistently abundant across several samples, with some samples displaying a relative abundance approaching 0.75. This suggests that this parasitic group may be widespread in the pangolin population. *Eimeria* is a common intestinal parasite that infects both wild and domesticated mammals, primarily transmitted through the fecal-oral route. It is known to cause clinical symptoms such as diarrhea, dehydration, and mucosal damage. Although Gregarina primarily parasitizes invertebrates, the detection results indicate a significant increase in the abundance of Gregarina in certain samples, suggesting that pangolins may carry Gregarina. It is worth noting that while Gregarina typically does not infect mammals, some species in this genus possess the potential for cross-host transmission within ecosystems. This may indicate a risk of parasite transmission between individual pangolins.

## 4 Discussion

This study systematically analyzed the composition and diversity characteristics of gastrointestinal parasites in the *M. pentadactyla* and the *M. javanica* using high-throughput amplicon sequencing technology. The results revealed that both pangolin species harbored dominant groups, such as Apicomplexa and Nematoda. Additionally, significant differences in parasite composition were observed between species and across individuals of different sexes.

Pangolins primarily feed on ants and termites by digging into the soil (21), and this specialized feeding behavior leads to frequent contact with the soil and its potential pathogens (22), thus exposing them to the risk of various parasitic infections. These parasites, as disease pathogens, pose a potential threat to the health of the host (23). In the humid and hot ecosystems of southern China, the favorable temperature and humidity conditions facilitate the survival and transmission of parasites, thereby increasing the likelihood of parasitic infections (24). The *Eimeria* (25), Acanthocephala (26), and Nematodes (27) detected in this study are primarily transmitted through fecal-oral or transdermal routes (28). These transmission pathways are closely associated with the host’s natural behaviors, such as foraging, drinking, and soil contact in their habitat (29).

Additionally, our study identified protists with potential pathogenicity, such as *Eimeria* (30), *Cryptosporidium* (31), and other free-living protozoa, including *Acanthamoeba* (51), *Colpoda*, *Vermamoeba*, *Bicosoecida*, and *Trinema*. But no direct evidence of infection has been confirmed in pangolins. To date, studies have indicated that pangolins can be infected with *Eimeria* (32). The main taxa identified in this study are summarized in Table 1. Cryptosporidia are intracellular protozoan parasites that are zoonotic. Infection with Cryptosporidia often leads to clinical symptoms in the host, such as diarrhea, anorexia, fever, and malabsorption (33). *Acanthamoeba* is not only a major pathogen responsible for diseases such as *Acanthamoeba* keratitis and granulomatous amoebic encephalitis, but it can also cause severe infections in organs such as the brain, lungs, and skin. These infections can lead to tissue damage and, in severe cases, result in host mortality (34). Furthermore, this study found that some individuals were infected with parasitic nematodes, including *Ancylostoma* and *Strongyloides* (35), which can cause systemic symptoms such as anemia and malabsorption, severely affecting the host’s overall health. The phylum Acanthocephala was also detected in this study. These parasites primarily penetrate the intestinal epithelium through their hooked proboscis, causing local tissue damage in the host (36). Notably, reports on Acanthocephala are exceedingly rare, with the only available pathological report being from Birgit Sist et al., which described the infection of Acanthocephala in the black-bellied pangolin, leading to small intestine perforation and secondary peritonitis, ultimately resulting in the death of the animal (37). The identification of these pathogenic parasites enhances our understanding of the potential sources of infection within pangolins (Table 1).

**Table 1:** Parasitic species identified in pangolins through amplicon sequencing and their medical/veterinary relevance.

Species	Medical/veterinary relevance	Infection history in pangolins/mammals
Rhabditida	Infection with *Ancylostoma* typically manifests as abdominal pain, diarrhea, anemia, and weight loss, and may also present with dermatological and respiratory symptoms.	Previous studies have isolated *Ancylostoma* sp. from *M. javanica* (22).
	Infection with *Leipernema leiperi n.g., n.sp.* in mammals typically manifests as abdominal pain, diarrhea, weight loss, anemia, and potentially immune responses such as dermatitis, with possible respiratory symptoms if larvae migrate to the lungs.	Studies have indicated the discovery of *Leipernema leiperi n.g., n.sp.*, in *M. pentadactyla* (45).
	Strongylids spp. typically, parasitize the cecum and colon of mammals, often presenting symptoms such as diarrhea, weight loss, and anemia.	Studies have identified Strongylids spp. from the feces of wild pangolins (46).
*Eimeria*	Causes coccidiosis, with symptoms such as diarrhea, weight loss, and potentially death.	*Eimeria* has been identified in the *Phataginus tricuspis* and the *M. javanica* (32).
Acanthocephala	Parasitic worms that attach to the intestinal epithelium using a proboscis, causing localized tissue damage, hemorrhage, and necrosis.	Reported in *Phataginus tetradactyla*, leading to small intestine perforation and secondary peritonitis, resulting in the animal’s death (37).
*Cryptosporidium*	An intracellular protozoan parasite that is zoonotic, causing gastrointestinal symptoms like diarrhea, anorexia, fever, and malabsorption.	The study indicates that free-ranging dogs can serve as hosts for *Cryptosporidium* species (47).
*Acanthamoeba*	Infection with *Acanthamoeba* may manifest as ocular inflammation, neurological symptoms, respiratory infections, skin lesions, and generalized weakness.	Studies have shown that *Acanthamoeba* spp. are found in large quantities at the sites of gastric ulcers and perforations in anteaters (48).
*Colpoda*	*Colpoda* is a free-living ciliate protozoan primarily found in soil, and infection with this organism can lead to diarrhea.	Existing studies have shown that *Colpoda* has been detected in free-ranging ruminants, including yaks, Tibetan sheep, and Tibetan goats (49).
*Vermamoeba*	The genus *Vermamoeba* is a protozoan found in aquatic environments, soil, and air. In immunocompromised individuals, *Vermamoeba* can cause brain or disseminated infections.	*Vermamoeba* has been found to infect the gut of non-human primates (50).
*Bicosoecida*	*Bicosoecida* is an aquatic protozoan group, primarily consisting of free-living, non-pathogenic organisms.	No existing literature indicates that *Bicosoecida* can infect mammals; instead, they primarily function as planktonic organisms participating in the ecological cycle.
*Trinema*	*Trinema* is a free-living protozoan that inhabits aquatic environments or moist soils, and it is typically harmless.	There is no clear evidence in the literature suggesting that *Trinema* can infect mammals. They primarily inhabit aquatic environments or moist soils and exhibit no apparent parasitic behavior.
Gregarina	Gregarina is a protozoan parasite that primarily infects invertebrates. It does not directly infect mammals, but it may cause mild intestinal discomfort in its host.	Infection in pangolins and mammals has not yet been confirmed. Gregarina typically parasitizes the intestines or body cavities of insects, arthropods, and other invertebrates.

In this study, *Eimeria* was identified as a significant parasitic protozoan in the gastrointestinal tract, alongside Rhabditida, which were found to be important parasites in both pangolin species. Although free-living organisms, such as *Acanthamoeba*, were also detected, they should not be considered parasites. Although these organisms might occasionally interact with the host under certain environmental conditions, their pathogenicity is relatively weak, and they typically do not cause significant health issues (19). Adonis and PCoA analyses revealed significant differences in the parasite community structure; the relatively low *R*^2^ values suggest a weak explanatory power for these differences. This limitation may stem from the high variability in parasite communities, particularly between different host species and sexes. Furthermore, unaccounted factors such as host immune status, environmental conditions, and the diversity of community composition could have a substantial impact on the community structure, thereby influencing the explanatory power of the analytical models. Previous studies have established that host immune status is a critical determinant in shaping parasitic infections (38). For instance, differential immune responses to parasitic infections have been documented between brown trout and rainbow trout, which correspond to variations in their tolerance and resistance to parasites (39). Furthermore, environmental factors, including temperature, humidity, and vegetation type, substantially affect the transmission dynamics and diversity of parasites (40, 41). Lund and Bensch (42), for example, demonstrated that habitat alterations markedly influence the species composition and transmission patterns of avian blood parasites. Recent evidence further highlights a close interplay between host immune responses and energy metabolism, with immunometabolic regulation potentially modulating host–parasite interactions and infection outcomes. Specifically, Troha and Ayres (43) revealed that immune-mediated modulation of host metabolic pathways can reshape the physiological milieu in which parasites such as *Plasmodium* develop, thereby affecting disease dynamics and parasite periodicity. Additionally, host hormone levels are intricately linked to parasitic infections; a comprehensive meta-analysis showed that parasite infections provoke widespread elevations in glucocorticoid hormones in vertebrate hosts, which subsequently modulate immune function and influence host susceptibility to parasitic challenges (44).

In summary, this study not only clarified the composition and diversity of gastrointestinal parasites in *M. pentadactyla* and *M. javanica* but also confirmed the presence of Cryptosporidia and *Acanthamoeba* infections in pangolins. These findings offer a theoretical basis for understanding the interactions between pangolin hosts and their parasites, as well as for disease prevention and control, ultimately contributing to improving pangolin health and reducing the risk of disease transmission.

## 5 Conclusion

This study is the first to systematically analyze the gastrointestinal community composition and diversity of *M. pentadactyla* and *M. javanica* using high-throughput amplicon sequencing technology. Our findings revealed the presence of several potentially pathogenic parasitic and protozoan groups in both pangolin species. While this study provides important theoretical insights into the gastrointestinal parasitic communities of pangolins and their potential pathogenicity, the exploratory nature of the research means that the current results only reveal community composition and potential pathogenicity, without confirming the presence of active infections. Therefore, follow-up studies are needed to verify whether these parasites are actively infecting the hosts and to assess their clinical impact on pangolin health. Further research should include more precise methods to detect parasite activity to confirm whether these parasites pose a health threat to pangolins. Additionally, studies should focus on understanding the transmission risks and clinical implications of these parasites in the wild, as well as their potential impact on other species in the pangolins’ habitat. These studies will provide a more solid scientific foundation for the prevention, treatment strategies, and conservation measures aimed at reducing the negative effects of parasitic infections on pangolin populations.

## Data Availability

The datasets presented in this study can be found in online repositories. The names of the repository/repositories and accession number(s) can be found at: NCBI repository, Accession number: PRJNA1301566.
